# The Effects of Korean Parents’ Smartphone Addiction on Korean Children’s Smartphone Addiction: Moderating Effects of Children’s Gender and Age

**DOI:** 10.3390/ijerph18136685

**Published:** 2021-06-22

**Authors:** Hye-Gyeong Son, Heeran J. Cho, Kyu-Hyoung Jeong

**Affiliations:** 1 College of Nursing, Kosin University, Busan 49104, Korea; hkprin@kosin.ac.kr; 2 Department of Health Administration, Yonsei University, Seoul 03021, Korea; 3 Department of Social Welfare, Semyung University, Jecheon 27136, Korea; jqbrother@semyung.ac.kr

**Keywords:** addictive behavior, children and adolescents, mirror neuron, parental attitude, smartphone addiction

## Abstract

*Background*: Smartphone addiction among children and adolescents has a negative effect, as excessive use of smartphones can cause physical symptoms, such as fatigue, indigestion, and sleep problems, as well as psychopathological problems, such as depression, anxiety, and impulsiveness. *Materials and Methods*: This study was carried out using the Korean Children and Youth Panel Survey 2019 conducted by the National Youth Policy Institute. The total number of participants was 4656 youths (2290 in grade 5 and 2366 in grade 8), and the dependent and independent variables were smartphone addiction in children and parents, respectively. Multiple regression analysis was conducted by Stata 15.0 SE. *Results*: First, the level of parents’ smartphone addiction affected that of children and adolescents. Second, the children’s and adolescents’ age affected the level of smartphone addiction, while their gender did not affect it. Third, the relationship between the levels of parents’ and children’s smartphone addiction was not moderated by the genders and ages of the children and adolescents. *Conclusions*: It was confirmed that as parents’ smartphone addiction increased, that of children increased, and smartphone addiction was found to increase in the second grade of middle school (12 years old) rather than grade 5 of elementary school (10 years old). Parents’ and children’s smartphone addiction was not moderated by children’s and adolescents’ gender and age.

## 1. Introduction

### Necessity of Research

Due to the advancement of technology and the rapid development of the internet, the number of mobile phone users continues to increase every year. More than seven billion people worldwide use mobile phones and smartphones. People’s need for connectivity and internet usage climbed from about 6.5% to 43.0% worldwide between 2000 and 2015; there was a sevenfold increase [1]. With this phenomenon, smartphones have become indispensable for most young people [2,3], and smartphone addiction has also increased, especially among adolescents [4,5,6,7]. According to the 2019 survey on smartphone overdependence, one in three teenagers aged 10–19 was addicted to smartphones in South Korea at that time [8]. Adolescents at risk of overdependence on smartphones made up 30.2% of all South Koreans, adults (ages 20 to 59) made up 18.8%, and those in their 60s made up 14.9% [8]. Smartphones are essential for adolescence to express themselves and form or maintain relationships with others [9], but it was found that they feel serious anxiety and deprivation without a smartphone [10].

Smartphone addiction in children and adolescents has a negative effect, as excessive use of smartphones can cause physical symptoms, such as fatigue, indigestion, and sleep problems, as well as psychopathological problems, such as depression, anxiety, and impulsiveness [11,12,13,14]. In addition, Gönener et al. stated that each use of a smartphone could cause physiological changes, such as headache, dizziness, tinnitus, and an increase in body temperature [15]. Smartphone addiction can also be a problem socially, as it has a negative effect on social development [16], and it has the potential to negatively affect school life and academic achievement [17]. In addition, excessive smartphone use may cause unexpected and possibly exorbitant phone charges, which may cause economic problems for some families and conflicts with parents accordingly [18]. Furthermore, children and adolescents who are addicted to smartphones are not only unable to concentrate on their studies, but they also often show problems in forming friendships due to deviant behavior [19]. Moreover, as children spend more time immersed in relationships in the virtual world, they may experience difficulties forming and maintaining interpersonal relationships in the real world [18].

Adolescent smartphone addiction has been found to be different according to gender and age. In Finland, female adolescents used mobile phones more than male students [20], and in South Korea, female adolescents were more vulnerable to the risk of overdependence than male adolescents [21]. Additionally, female youths were at greater risk of smartphone overdependence than male youths in all age groups, excluding infants and children by gender [22]. In terms of age, it was also found that middle school students were the most vulnerable to the risk of overdependence of all school levels [21]. Furthermore, middle school students were higher in both high-risk and potential-risk groups than elementary and high school students [22].

Identifying factors that affect children’s smartphone addiction is significant in terms of preventing smartphone addiction. This study aims to focus on parents’ smartphone addiction as a factor influencing children’s smartphone addiction. While previous studies examining the relationship between parents’ and children’s smartphone addiction are insufficient due to a dearth of research on this issue, the significance of the relationship can be inferred by mirror neuron theory and social learning theory.

According to the mirror neuron theory, neurons activated as if performing another person’s behavior when they perceive that behavior is called ‘mirror neurons’ [23]. They convert the viewed behavior into a neuronal signal, and the other person’s mental state can be gleaned through ‘mirroring’ and empathized with it [24]. An analysis examining the correlation between parents and children in the 2016 survey on smartphone overdependence [25] found that when the parents are in the at-risk group, the percentage of adolescent children in the at-risk group is 36.0%, and the percentage of infants and children in the at-risk group is 23.5%. It was revealed that when the parents are in the at-risk group, the proportion of their children in the at-risk group reaches 60.0%. In particular, it has been found that if parents are obsessed with smartphones, demonstrated by behavior such as frequent habitual checking of smartphones and becoming anxious without smartphones, it can lead to problematic behavior in young children [26].

In addition, the phenomenon of smartphone addiction between parents and children can be investigated based on the social learning theory’s concept of ‘modeling’ for parents. Bandura argued that social learning theory states that human learning is accomplished simply by seeing the behavior of a certain model [27]. This means that behavior is acquired by imitating and observing the behavior of others within a social context. In behaviorist theory, one of several theories used to explain changes in learning and behavior, it is said that the imitation of learning and behavior occurs only through direct reinforcement and punishment. In social learning theory, it is said that learning is accomplished simply by looking at the behavior of the model without reinforcement and punishment. This is also called observational learning theory, modeling, or imitative learning. In addition, in the process of observing and learning the behavior of modeling, real models appeared to have the greatest impact, followed by media, such as movies and videos. As students are highly receptive to practical models, they are greatly influenced by teachers and parents, and learn more easily accordingly [28].

It was revealed that parents’ behaviors of using the internet or technology in their homes influenced the frequency of their children’s and adolescents’ internet and technology usage behavior, addictive usage habits, and related knowledge [29,30]. This effect would be much the same for smartphones. If parents use smartphones obsessively, it is highly likely that children will have easy access to smartphones and imitate their parents by also using smartphones obsessively.

In previous studies, factors influencing smartphone addiction in children and adolescents were studied in relation to the individual’s personality, disposition, and psychological factors [31,32,33,34], and regarding parental factors, the parenting attitudes of parents were mostly studied [35,36]. There are studies confirming the direct relationship between parents’ smartphone addiction and their children’s smartphone addiction, but they have mainly been focused on infants and toddlers [37,38]. Thus, this study aims to examine the effects of parents’ smartphone addiction on smartphone addiction among school-aged children and adolescents and to verify the moderating effects of children’s and adolescents’ gender and age.

## 2. Methods

### 2.1. Research Model

**Figure 1:**
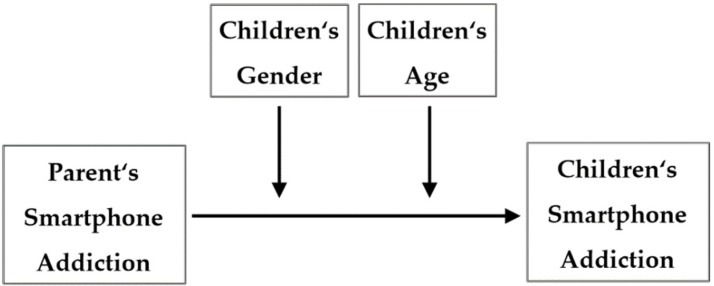
Research model.

The purpose of this study is to examine the effects of parents’ smartphone addiction on that of their elementary- and middle-school-aged children and the moderating effects of gender and age. The study model was set up as shown in Figure 1.

### 2.2. Data

This study was conducted using the data from the second survey (2019) of the Korean Children and Youth Panel Survey (KCYPS), conducted by the National Youth Policy Institute (NYPI). The Korean Children and Youth Panel Survey is a representative survey of children and adolescents in South Korea. It provides basic data for policy establishment and academic research related to children and adolescents by constructing panel data that can comprehensively grasp changes in their growth and development. The sample population for the KCYPS was extracted by a multi-tiered colony sampling method based on the population of students in the fifth grade of elementary school (grade 5) and students in the second year of middle school (grade 8) nationwide collected during the first survey (2018), and a total of 5197 respondents completed the survey. This study used data from only grade 5 and grade 8 children, as the original data included these two grades to briefly compare and contrast children and adolescents (2607 students in grade 5 and 2590 students in grade 8). In this study, the second survey respondents (2019) were used as the subjects, and 4656 participants (2290 in grade 5 and 2366 in grade 8) with no missing values in the major variables were analyzed.

### 2.3. Measures

#### 2.3.1. Dependent Variable and Independent Variable

The dependent and independent variables of this study were smartphone addiction of children and parents, respectively, and the KCYPS was used by referring to the Smartphone Addiction Proneness Scale (SAPS) developed by Kim [38]. The SAPS consists of a total of 15 questions and makes use of a 4-point scale (strongly disagree = 1, disagree = 2, agree = 3, strongly agree = 4) (Table 1). Out of 15 questions, the following were reverse-coded: ‘using a smartphone does not interfere with what you are doing (studying)’, ‘I am not anxious without a smartphone’, and ‘I don’t spend a lot of time using a smartphone’, and the average of the questions was calculated. The higher the score, the higher the smartphone addiction score was interpreted. In Kim et al.’s study, the reliability of SAPS was verified with a Cronbach’s alpha of 0.88 [39], and in this study, Cronbach’s alpha was 0.85, showing similarly high reliability.

#### 2.3.2. Moderator Variables

The moderator variables used in this study are gender (male = 0, female = 1) and age (grade 5 = 0, grade 8 = 1).

#### 2.3.3. Statistical Analyses

Stata 15.0 SE was used for data analysis. First, a descriptive statistical analysis was conducted to confirm the level of use of smartphones by children and parents for each purpose. Second, an independent t-test was conducted to confirm the differences in major variables according to the children’s gender and age. Third, multiple regression analysis was conducted to verify the moderating effect of the children’s gender and age in the relationship between parents’ smartphone addiction and that of children. In addition, a bootstrap procedure was conducted 5000 times with a 95% confidence interval to examine each moderation effect.

## 3. Results

### 3.1. Level of Smartphone Usage by Children and Parents by Purpose

The level of smartphone usage by children and parents for each purpose of using smartphones was measured on a 4-point Likert scale (1 = never use, 2 = rarely use, 3 = sometimes use, 4 = frequently use) (Table 2). Among the purposes of using smartphones for both children and parents, phone calls with family members were the highest for both parents and children at 3.46 points (SD = 0.68) and 3.64 points (SD = 0.56), respectively. In the case of children, text messages with friends (M = 3.44, SD = 0.79) and watching TV and videos (M = 3.36, SD = 0.86) were relatively high. In the case of parents, text messages with family members (M = 3.53, SD =0.60) and information retrieval (M = 3.49, SD = 0.70) were relatively high, showing divergence from the children’s uses.

### 3.2. Differences in Main Variables According to Gender and Age

Table 3 and Table 4 show the results of the independent t-test to determine the differences in the major variables according to the children’s gender and age. First, there was a significant difference in the parents’ smartphone addiction scores according to the children’s gender (t = −3.146, *p* < *0*.01). Parents’ smartphone addiction compared with that of boys was 1.87 points (SD = 0.42) on average, and parents’ smartphone addiction compared with that of girls was 1.91 points (SD = 0.44) on average, indicating that parents’ smartphone addiction compared with that of girls was statistically significantly higher than parents’ smartphone addiction compared with that of boys. However, there was no significant difference in children’s smartphone addiction according to the gender of the children. In the case of children’s ages, there was a significant difference in children’s smartphone addiction (t = −10.869, *p* < *0*.001). Specifically, children’s smartphone addiction was significantly lower in grade 5 (M = 2.00, SD = 0.47) than in the grade 8 (M = 2.14, SD = 0.44). On the other hand, parents’ smartphone addiction scores did not show any significant difference according to the age of the children. 

### 3.3. Analysis of Research Model

Table 5 shows the moderating effect of children’s genders and ages in the relationship between parents’ smartphone addiction and that of children. The regression equation is statistically significant (F = 108.22, *p* < *0*.001). When examining the analysis results in detail, it was confirmed that the independent variables of parents’ smartphone addiction (B = 0.307, *p* < *0*.001) and children’s ages (B = 0.237, *p* < *0*.001) had a significant effect on children’s smartphone addiction. That is, the higher the parents’ smartphone addiction scores, the higher those of children. This relationship was even more pronounced for children in grade 8 than those in grade 5. Meanwhile, it was confirmed that there was no significant interaction between smartphone addiction and the children’s age. In addition, it was confirmed that parents’ smartphone addiction (A) × children’s gender and parents’ smartphone addiction (A) × children’s age did not have a significant effect. That is, the moderating effect of children’s genders and ages did not appear in the relationship between the levels of parents’ smartphone addiction and those of children.

## 4. Discussion

This study examined the effects of parents’ smartphone addiction on that of children and the moderating effects of children’s gender and age using data from the Korean Children and Youth Panel. The main research results are as follows.

First, it was found that the level of parents’ smartphone addiction affected that of children. This result is in line with the finding of Bonetti, Campbell, and Gilmore that parents’ attitudes towards smartphones or smartphone usage behaviors influenced their children’s smartphone addiction rate [40].

Second, the children’s age affected their smartphone addiction scores, while their gender did not. When parents were addicted to smartphones, grade 8 students were more addicted to smartphones than grade 5 students. This result conflicts with a study conducted using parents who were not addicted to smartphones; in that instance, it was found that because younger children’s mental development is not complete, they are easily captivated by smartphones and are likely to become addicted to them [41]. It is speculated that children whose parents are addicted to smartphones are exposed to regular and persistent smartphone use at home for a longer duration, so they become more addicted as they get older.

Third, the genders and ages of children did not moderate the relationship between parents’ and children’s smartphone addiction scores. While previous studies have confirmed that the gender and age of children affect smartphone addiction [20,21,22,39], this study shows that children’s gender and age may not have a significant effect as moderator variables. Furthermore, although parental smartphone addiction has a strong effect on that of children, the effect does not change according to the children’s gender and age. The results of this study are meaningful, as the relationship between gender and age of children and smartphone addiction is studied from a different dimension.

## 5. Conclusions

As smartphone addiction is a social issue in South Korea that is becoming more pressing over time, governmental bodies should consider intervening by conducting extensive research regarding adolescents’ smartphone addiction and allocating funds for managing consultation centers. As parents’ smartphone addiction significantly affects that of their children, parents also need to be aware of the significance of the problems that smartphones pose. For interventions with children, it is essential to consider the age of the children. For instance, more active intervention is necessary for middle school students, and public education for middle school students to prevent smartphone addiction can be crucial. As gender does not have a significant effect, education can be delivered to both genders at once. As the moderating effects between parents’ and children’s smartphone addiction are not significant, children’s gender and age do not need to be considered when intervening in children’s smartphone addiction caused by parental smartphone addiction.

## 6. Limitations

There are few limitations, as this study analyzed secondary data. Specifically, all age groups could not be included in the analysis, and the ages of children are limited to the fifth year of elementary school and the second year of middle school, as data for all age groups were not available. As a result, it was difficult to grasp the moderating effect of age properly. Therefore, it is essential for subsequent studies to further refine the effect through a categorized analysis according to the gender or age of parents and to include all age groups in elementary and middle schools for analysis.

## Figures and Tables

**Table 1:** Smartphone addiction proneness scale.

Items
My school grades dropped due to excessive smartphone use
2. I have a hard time doing what I have planned (study, do homework, or go to after school classes) due to using smartphone
3. People frequently comment on my excessive smartphone use
4. Family or friends complain that I use my smartphone too much
5. My smartphone does not distract me from my studies
6. Using a smartphone is more enjoyable than spending time with family or friends
7. When I cannot use a smartphone, I feel like I have lost the entire world
8. It would be painful if I am not allowed to use a smartphone
9. I get restless and nervous when I am without a smartphone
10. I am not anxious even when I am without a smartphone
11. I panic when I cannot use my smartphone
12. I try cutting my smartphone usage time, but I fail
13. Even when I think I should stop, I continue to use my smartphone too much
14. Spending a lot of time on my smartphone has become a habit
15. I don’t spend a lot of time using smartphone

**Table 2:** Level of smartphone usage by children and parents by purpose.

Item	Children (*n* = 4656)		Parents (*n* = 4656)	
	M	SD	M	SD
Phone call with family	3.46	0.68	3.64	0.56
Text message with family	3.23	0.77	3.53	0.60
Phone call with a friend	3.30	0.78	3.19	0.65
Text message with a friend	3.44	0.79	3.32	0.67
Use of SNS	2.55	1.20	2.18	1.05
Game	3.01	0.97	1.86	0.99
Photo/video shooting	2.96	0.86	3.26	0.66
Watching TV and videos	3.36	0.86	2.70	0.98
Listening to music	3.17	0.90	2.96	0.87
Information retrieval	2.98	0.88	3.49	0.70
Document view	2.52	1.12	2.32	0.98
Academic or work related	2.47	0.95	2.76	0.96

Note: *n* = sample size, M = mean, SD = standard deviation.

**Table 3:** Differences in the main variables by children’s gender.

Variables	Male (*n* = 2414)		Female (*n* = 2242)		t
	M	SD	M	SD	
Parents’ smartphone addiction	1.87	0.42	1.91	0.44	−3.146 **
Children’s smartphone addiction	2.07	0.45	2.07	0.47	−0.680

Note: *n* = sample size, M = mean, SD = standard deviation, t = t-statistic, ** *p* < 0.01.

**Table 4:** Differences in the main variables by children’s age.

Variables	Grade 5 (*n* = 2290)		Grade 8 (*n* = 2366)		t
	M	SD	M	SD	
Parents’ smartphone addiction	1.90	0.43	1.87	0.43	1.631
Children’s smartphone addiction	2.00	0.47	2.14	0.44	−10.869 ***

Note: *n*= sample size, M = mean, SD = standard deviation, t = t-statistic. *** *p* < 0.001.

**Table 5:** Research model analysis.

Variables		B	S.E.
Independent variable	Parents’ smartphone addiction (A)	0.307 ***	0.026
Moderator variable	Children’s gender (ref. male) (B)	−0.063	0.058
	Children’s age (ref. grade 5) (C)	0.237 ***	0.058
Interaction	A × B	0.035	0.030
	A × C	−0.045	0.030
constant		1.411 ***	0.051
R^2^		0.124	
F (sig.)		108.22 ***	

Note: B = B-statistic; S.E. = standard error, sig. = significance; *** *p* < 0.001.

## Data Availability

Not applicable.
